# Mass Spectrometry Applications in Biomedical Research

**DOI:** 10.1155/2015/827370

**Published:** 2015-05-21

**Authors:** Fa-Yun Che, Hai-Teng Deng, Shi-Jian Ding

**Affiliations:** ^1^Department of Pathology, Albert Einstein College of Medicine, Bronx, NY 10461, USA; ^2^School of Life Sciences, Tsinghua University, Beijing, China; ^3^Diabetes and Obesity Research Center, Sanford-Burnham Medical Research Institute at Lake Nona, Orlando, FL 32827, USA

We are so happy and excited to see our special issue published. With the tremendous advances of modern mass spectrometry (MS) techniques, MS has become one of the essential analytical tools to biological and biomedical research. Mass spectrometry provides rapid and sensitive qualitative and quantitative analysis of biomolecules (proteins, peptides, oligosaccharides, lipids, DNA, and RNA), drugs, and metabolites. In this special issue we have collected 12 original research articles and 2 review articles. MS has been successfully applied to high-throughput proteome-wide analysis of proteins, protein-protein interactions, and protein posttranslational modifications (PTMs) in cells, tissues, or organs. L. Jin et al. reported phosphoproteomic analysis of gossypol-induced apoptosis in ovarian cancer cell line. In their work, about 9750 phosphopeptides from 3030 phosphoproteins were identified. They found gossypol induced upregulation of LATS1 which phosphorylates YAP1. T. Guo et al., using 2DGE-based Western blotting and MS/MS technique, identified 24 glioblastoma tyrosine-phosphorylated proteins. R. Xu et al. reported the application of SILAC-based quantitative proteomic analysis to myeloma cells. They found gossypol treatment induced ROS production and cell necrosis. Proteomics showed that gossypol induced changes in expression levels of 585 proteins including down-regulation of DNA repair proteins, and upregulat ion of death-associated factors. In another quantitative proteomic work performed by Q. Xiong et al., tandem mass tag (TMT) labeling approach was employed to comprehensively compare the differential expression of proteins in receptor activator of nuclear factor-*κ*B ligand- (RANKL-) induced osteoclasts in the presence and absence of estrogen. In the past several years, we have also witnessed fast growth in metabolomics fueled by MS analysis. The wide implementation of MS certainly has resulted in and will continuously benefit advancement in-depth understanding of biological processes and their molecular mechanisms underlying. In terms of biomarker discovery for many human diseases and therapeutics, MS-based proteomics and metabolomics are playing an important role and the application of MS to clinical molecular diagnosis will be expected in the near future. A. Zhang et al. reviewed the metabolomics for biomarker discovery and highlighted the importance and benefit of metabolomics for identifying biomarkers. C.-T. Chang et al. presented a review on recent developments in MS-based quantitative proteomics techniques, high-density lipoprotein (HDL) proteomics, and lipoprotein modification in biomarker discovery for atherosclerotic vascular disease. V. Tambor's group has explored the potential peripartum markers of infectious-inflammatory complication in preterm birth. Y. Cheng et al. reported that they implemented a combination of peptide ligand library beads and 1D Gel-LC-MS/MS to serum samples from patients with ovarian cancer and from healthy controls. Their proteomic data suggested that RBP4 protein is a potential biomarker of ovarian cancer. Their ELISA and immunohistochemistry experiments with RBP4 confirmed the proteomic results. T. Ai's group presented their study on serum peptide marker discovery for gestational diabetes mellitus (GDM). MS has been widely used to perform pharmacokinetics studies of the effects of biological systems in a determined drug. Here, M. Sánchez-Sierra et al. described MS in pharmacokinetic studies of a synthetic compound for spinal cord injury treatment. L. Godderis et al. presented another topic which is MS application on analysis of DNA methylation and/or hydroxymethylation. They described a LC-MS/MS method to quantify and compare simultaneously global methylation and hydroxymethylation in human DNA of different tissues. C. Gao et al. presented a comparative study on* Nell-1* hypermethylation levels among tumor, paracarcinoma, and normal tissues from gastric cancer patients. They found a significant difference in DNA methylation status between gastric cancer or paracarcinoma and normal tissue. Another topic in this special issue is presented by H. Wang's group, in which they described their successful application of LC-MS to the rapid differentiation of *β*-lactam and ring-opened *β*-lactam impurities in cefixime, cefdinir, and cefaclor antibiotics.

As Guest Editors of this special issue, we hope to see in the future that there are more and more researchers to present their work in this special issue in this peer-reviewed journal to exchange, promote, and culture our scientific spirits and to accelerate the advance of mass spectrometry itself and its application in biomedical research and clinical diagnosis for the benefits of human.

*Fa-Yun Che* *Hai-Teng Deng* *Shi-Jian Ding*

